# Diversity of *Streptococcus* spp. and genomic characteristics of *Streptococcus uberis* isolated from clinical mastitis of cattle in Bangladesh

**DOI:** 10.3389/fvets.2023.1198393

**Published:** 2023-07-18

**Authors:** Jayedul Hassan, Md. Abdus Sattar Bag, Md. Wohab Ali, Ajran Kabir, M. Nazmul Hoque, Muhammad Maqsud Hossain, Md. Tanvir Rahman, Md. Shafiqul Islam, Md. Shahidur Rahman Khan

**Affiliations:** ^1^Department of Microbiology and Hygiene, Faculty of Veterinary Science, Bangladesh Agricultural University, Mymensingh, Bangladesh; ^2^Department of Gynecology, Obstetrics and Reproductive Health, Faculty of Veterinary Medicine and Animal Science, Bangabandhu Sheikh Mujibur Rahman Agricultural University, Gazipur, Bangladesh; ^3^Department of Biochemistry and Microbiology, North South University, Dhaka, Bangladesh; ^4^NSU Genome Research Institute (NGRI), North South University, Dhaka, Bangladesh

**Keywords:** *Streptococcus uberis*, diversity, clinical mastitis, cattle, WGS

## Abstract

**Introduction:**

Streptococci are the major etiology in mastitis in dairy cattle, a cause of huge economic losses in the dairy industries. This study was aimed to determine the diversity of *Streptococcus* spp. isolated from clinical mastitis of cattle reared in Bangladesh.

**Methods:**

A total of 843 lactating cattle reared in four prominent dairy farms and one dairy community were purposively included in this study where 80 cattle were positive to clinical mastitis (CM) based on gross changes in the udder (redness, swelling, and sensitive udder) and/or milk (flakes and/or clots). Milk samples were collected from all the eighty cattle with clinical mastitis (CCM) and twenty five apparently healthy cattle (AHC). Samples were enriched in Luria Bertani broth (LB) and one hundred microliter of the enrichment culture was spread onto selective media for the isolation of *Staphylococcus* spp., *Streptococcus* spp., *Enterococcus* spp., *Escherichia coli* and *Corynebacterium* spp., the major pathogen associated with mastitis. Isolates recovered from culture were further confirmed by species specific PCR.

**Results and Discussion:**

Out of 105 samples examined 56.2% (59/105), 17.14% (18/105), 9.52% (10/105) and 22.9% (24/105) samples were positive for *Staphylococcus*, *Streptococcus*, *Enterococcus faecalis* and *E. coli*, respectively. This study was then directed to the determination of diversity of *Streptococcus* spp. through the sequencing of *16S rRNA*. A total of eighteen of the samples from CCM (22.5%) but none from the AHC were positive for *Streptococcus* spp. by cultural and molecular examination. Sequencing and phylogenetic analysis of *16S rRNA* identified 55.6, 33.3, 5.6 and 5.6% of the *Streptococcus* isolates as *Streptococcus uberis*, *Streptococcus agalactiae*, *Streptococcus hyovaginalis* and *Streptococcus urinalis*, respectively. Considering the high prevalence and worldwide increasing trend of *S. uberis* in mastitis, in-depth molecular characterization of *S. uberis* was performed through whole genome sequencing. Five of the *S. uberis* strain isolated in this study were subjected to WGS and on analysis two novel ST types of *S. uberis* were identified, indicating the presence of at least two different genotypes of *S. uberis* in the study areas. On virulence profiling, all the isolates harbored at least 35 virulence and putative virulence genes probably associated with intramammary infection (IMI) indicating all the *S. uberis* isolated in this study are potential mastitis pathogen. Overall findings suggest that *Streptococcus* encountered in bovine mastitis is diverse and *S. uberis* might be predominantly associated with CM in the study areas. The *S. uberis* genome carries an array of putative virulence factors that need to be investigated genotypically and phenotypically to identify a specific trait governing the virulence and fitness of this bacterium. Moreover, the genomic information could be used for the development of new genomic tools for virulence gene profiling of *S. uberis*.

## Introduction

Bovine mastitis is the major hindering factor in the dairy industry responsible for huge economic losses worldwide. Bovine mastitis was estimated to cost €16–26 billion *per annum* worldwide (1) and contributed to the reduction of milk yield and quality, culling cows and veterinary care (2). Bacteria are the main cause of this disease among 130 well-known pathogens of bovine mastitis (3). Genus *Streptococcus* is major among the bacterial pathogens and account for 23%–50% of the total mastitis cases worldwide (4). Mastitis is classified into three distinct forms based on the degree of inflammation, sub-clinical, clinical and chronic (5), and the occurrence of *Streptococcus* have been reported in all three forms of this disease (6–8).

*Streptococcus agalactiae*, *Streptococcus dysagalactiae*, and *Streptococcus uberis* are most commonly responsible for bovine mastitis with varying global frequencies among *Streptococcus* spp. (4, 9). *S. agalactiae* was found in 90% of mastitis cases in the past; however, due to their contagious nature, the incidence of *S. agalactiae* was drastically reduced by implementing a five-point hygiene plan in the 1960s (3, 10, 11). In contrast, the majority of the causes of mastitis cases shifted towards environmental mastitis (3, 12). As a result, the prevalence of environmental mastitis pathogens such as *S. uberis* has increased remarkably (4, 13). However, the prevalence of the major streptococci was variable based on geographical locations (4). In Bangladesh, information on *Streptococcus* and their antibiotic susceptibility in mastitis are minimal (14–16), and to the best of our knowledge, no research has been documented on the occurrence, diversity and molecular characteristics of *Streptococcus* in clinical mastitis of cattle in Bangladesh. In Bangladesh, dairying is a growing industry and mostly dominated by small holding farms producing 70-80% of the country’s total milk (17). In recent years, dairy farming is going intensification and new large scales farm units (farms with ⁓200 dairy cows) are emerging; however, most of the large dairy farms belonged to the government and private sectors.^1^ Although mastitis is frequently reported from different corner of the country, reaching them and collecting information on therapeutic interventions are quite difficult and lacks authentications. Thus, the present study aimed to determine the diversity of *Streptococcus* spp. in clinical mastitis of cattle in the major dairy production units (farms/community) in Bangladesh and their antibiotic susceptibility pattern. In addition, the research was further extended to the genomic characterization and virulence profiling of the *S. uberis* isolated in this study through whole genome sequencing and subsequent bioinformatic analysis considering the increased prevalence of this pathogen in bovine mastitis.

## Materials and methods

### Ethical statement

This study was approved by the Animal Welfare and Experimentation Ethics Committee (AWEEC), Bangladesh Agricultural University, Mymensingh-2,202, Bangladesh [Approval No. AWEEC/BAU/2020(45)]. In addition, verbal as well as written consent of the farm owner or respective authority was taken before collecting samples.

### Sample collection and enrichment

Milk samples were collected from cattle with clinical mastitis (CCM) from four major dairy farms and a community (consisting several small holding dairy farms) at different locations in Bangladesh during the period from January 2019 to June 2021. Dairy farms and the community was purposively selected as they were well managed and major dairy units located in distinct geographical locations including Dhaka (23.8105° N, 90.3372° E), Mymensingh (24.7539° N, 90.4073° E) and Sirajganj (24.3141° N, 89.5700° E) districts of Bangladesh. A total of 2,731 dairy cattle comprising 843 lactating cows were included in this study (Supplementary Table S1) and clinical mastitis was diagnosed by the field or residential veterinarian through the observation of gross changes in the udder (redness, swelling, and sensitive udder) and/or milk (flakes and/or clots). A total of 105 milk samples comprising 80 from the cattle with clinical mastitis (CCM) and 25 from apparently healthy cattle (AHC) were collected at the study period. Strict hygienic conditions including sterile hand gloves, collection tubes [50 mL Falcon tubes (SPL Life Sciences, Gyeonggi-do, Korea)], were used during sample collection. In addition, the udder was cleaned with potable water, wiped with paper towel until the fecal materials completely removed and care was taken to avoid contact of the collection tube with the skin and/or teat of the mammary gland during sample collection. Furthermore, first few squirts of milk from the udder were discarded before collecting the original sample. A 10 mL composite milk sample was collected directly from the udder of a cow and carried to the laboratory in ice box for microbiological analysis. In the laboratory, 0.5 mL of the milk was enriched in 4.5 mL LB broth overnight at 37°C following the procedure described earlier by different authors (18–20).

### Isolation and identification of bacteria

One hundred microlitres of enriched culture was spread onto different selective media such as Mannitol salt agar (Himedia, Thane, Maharashtra, India) for *Staphylococcus* spp., modified Edwards medium (MEM) (Himedia) for *Streptococcus* and *Enterococcus* spp., MacConkey agar (Liofilchem, Roseto d. Abruzzi, Italy) for *Escherichia coli*, and brain heart infusion agar (Himedia) supplemented with 0.25 g/L potassium tellurite (Himedia) and 1.0% Tween 80 (Smart Lab, Tangerang, Indonesia) for *Corynebacterium* spp. After an overnight incubation at 37°C at least 10 colonies characteristic of the respective bacterium were screened by Gram’s staining followed by purification to single isolated colonies with uniform morphologies by repetitive culture onto selective media and Gram’s staining. Genomic DNA was extracted from the isolated colonies using the boiling method (21) and confirmed by species specific PCR for *Staphylococcus* spp., *Streptococcus* spp. (*S. agalactiae*, *S. dysagalactiae* and *S. uberis*), *Enterococcus* spp. (*E. faecalis*, *E. faecium*), *E. coli* and *Corynebacterium* spp. (22–28). Primers and PCR conditions followed for the identification of bacterial species are enlisted in the Supplementary Table S2. Streptococcus isolates were subjected to amplification and sequencing of *16S rRNA* using 8F (5′-AGAGTTTGATCMTGGC-3′) and 1492R (5′-TACCTTG TTACGACTT-3′) primers to further confirmation and discrimination (29). Briefly, amplification of *16S rRNA* was performed in 50 μL volume with 25 μL of 2X Go Taq^®^ G2 Green Mater Mix (Promega, WI, United States), 2.5 μL of each primer (10 pmol/μL) and 3 μL of DNA template. The PCR product was sent for sequencing commercially (Macrogen, Seoul, Korea). Alignment and phylogenetic analysis of the *16S rRNA* sequences were performed on CLC genomic workbench 22 (Qiagen, Germany).

### Antibiotic susceptibility testing of *Streptococcus* spp.

Susceptibility of *Streptococcus* spp. against antibiotics commonly used in mastitis treatment was performed by the disc diffusion method (30). A total of 14 antimicrobials of different classes were purchased and stored according to the manufacturer’s instructions before use (Oxoid, United Kingdom). The antimicrobials includes β-lactams [amoxicillin 10 μg (AMX), ampicillin 10 μg (AMP), ceftazidime 30 μg (CAZ), ceftriaxone 30 μg (CTR), cephalexin 30 μg (CN), and penicillin G 10 μg (P)], aminoglycosides [kanamycin 30 μg (K), gentamicin 30 μg (GEN), and streptomycin 10 μg (S), glycopeptides (vancomycin 30 μg (VA)], macrolides [azithromycin 15 μg (AZM)], phenicols [chloramphenicol 30 μg (C)], polypeptides [bacitracin 10 units (B)], and tetracyclines [tetracycline 30 μg (TE)] was used in this study. *E. coli* strain ATCC25922 was used as the control strain and the results were interpreted according to the Clinical and Laboratory Standards Institute (CLSI) guidelines (31). Isolates exhibiting resistance to three or more antibiotic classes were considered as multidrug-resistant (MDR) (32).

### Whole genome sequencing and annotation

Five of ten *S. uberis* isolated in this study were subjected to whole genome sequencing (WGS). For isolate selection no definite criteria were followed; however, from each *S. uberis* positive farm at least one isolate was selected for WGS (Supplementary Table S3). WGS was performed on an Illumina Nextseq 550 platform (Illumina, San Diego, CA, United States) in the commercial facility of Invent Technologies Ltd., Dhaka, Bangladesh. Briefly, pure isolated colony from LB agar plate was inoculated in LB broth and incubated overnight at 37°C. Genomic DNA was extracted from the overnight culture using QIAamp DNA Mini Kit (QIAGEN, Hilden, Germany) and the quantity was measured with a NanoDrop 2000 UV–Vis Spectrophotometer (Thermo Fisher, Waltham, MA, United States). Libraries were prepared from 1 ng of DNA using Nextera^™^ DNA Flex Library Prep Kit (Illumina, San Diego, United States) for short-read sequencing. Libraries were cleaned (AMPure XP beads; Beckman Coulter) and sequencing was performed on an Illumina Nextseq 550 platform (Illumina, CA, United States) using a 150 bp pairedend sequencing protocol. Quality of the FASTQ reads and trimming of low-quality residues was performed on Trimmomatic (Galaxy Version 0.38). Trimmed reads were assembled on a hybrid assembler Unicycler (Galaxy Version 0.4.8.0). Assembled contigs were annotated using Prokka (Galaxy Version 1.14.6), RAST (Rapid Annotation using Subsystem Technology),^2^ and NCBI prokaryotic genome annotation pipeline (PGAP) to identify the functional features. The annotated data were used for antibiotic resistance gene profiling, plasmid identification, sequence typing and virulence genes profiling.

Antibiotic resistance genes were identified using the Comprehensive Antibiotic Resistance Database (CARD),^3^ and for virulence gene detection, the *S. uberis* Putative Virulence Database suPVDB (33) and virulence factor database (VFDB)^4^ was explored. Further, the known virulence related genes (34, 35); were identified through search on NCBI blastn [Nucleotide BLAST: Search nucleotide databases using a nucleotide query (nih.gov)] and blastx [blastx: search protein databases using a translated nucleotide query (nih.gov)] platforms.

Average nucleotide identity (ANI) of the *S. uberis* sequences along with genome assemblies downloaded from the NCBI [*Streptococcus uberis*—Assembly—NCBI (nih.gov)] was calculated using a perl program GET_HOMOLOGUES (36). The genome sequences were further analyzed using the Roary pan genome pipeline (37) to identify orthologous genes in *S. uberis* and construction of a tree by clustering the core and accessory genes in a matrix. In addition, core genome based whole genome phylogeny and tree visualization were performed on CLC Genomic workbench (Qiagen) and iTOL v6 [iTOL: Interactive Tree Of Life (embl.de)], respectively. The comparative genomic feature of the study strains against the *S. uberis* strain 0140J (GenBank accession no.: AM946015.1) reference genome was visualized using BLAST Ring Image Generator (BRIG) version 0.95 (38). Pathways and subsystems for the strains under study were obtained from the Kyoto Encyclopedia of Genes and Genomes orthology (KEGG) (39), and the SEED databases (40), respectively. CRISPRCasFinder 4.2.20 software at Proksee tools;^5^ was used for rapid identification and classification of clustered regularly interspaced short palindromic Repeats (CRISPRs) and the PHASTER web server^6^ was used to identify a prophage sequence. The *S. uberis* genome sequences were examined for their sequence type (ST) through PubMLST website.^7^ Concatenated ST sequences were downloaded from PubMLST^8^ and the minimum spanning tree was constructed using the goeBurst algorithm of Phyloviz^9^ to identify the closest neighbors.

## Results

### Occurrence and diversity of *Streptococcus* spp.

**Figure 1:**
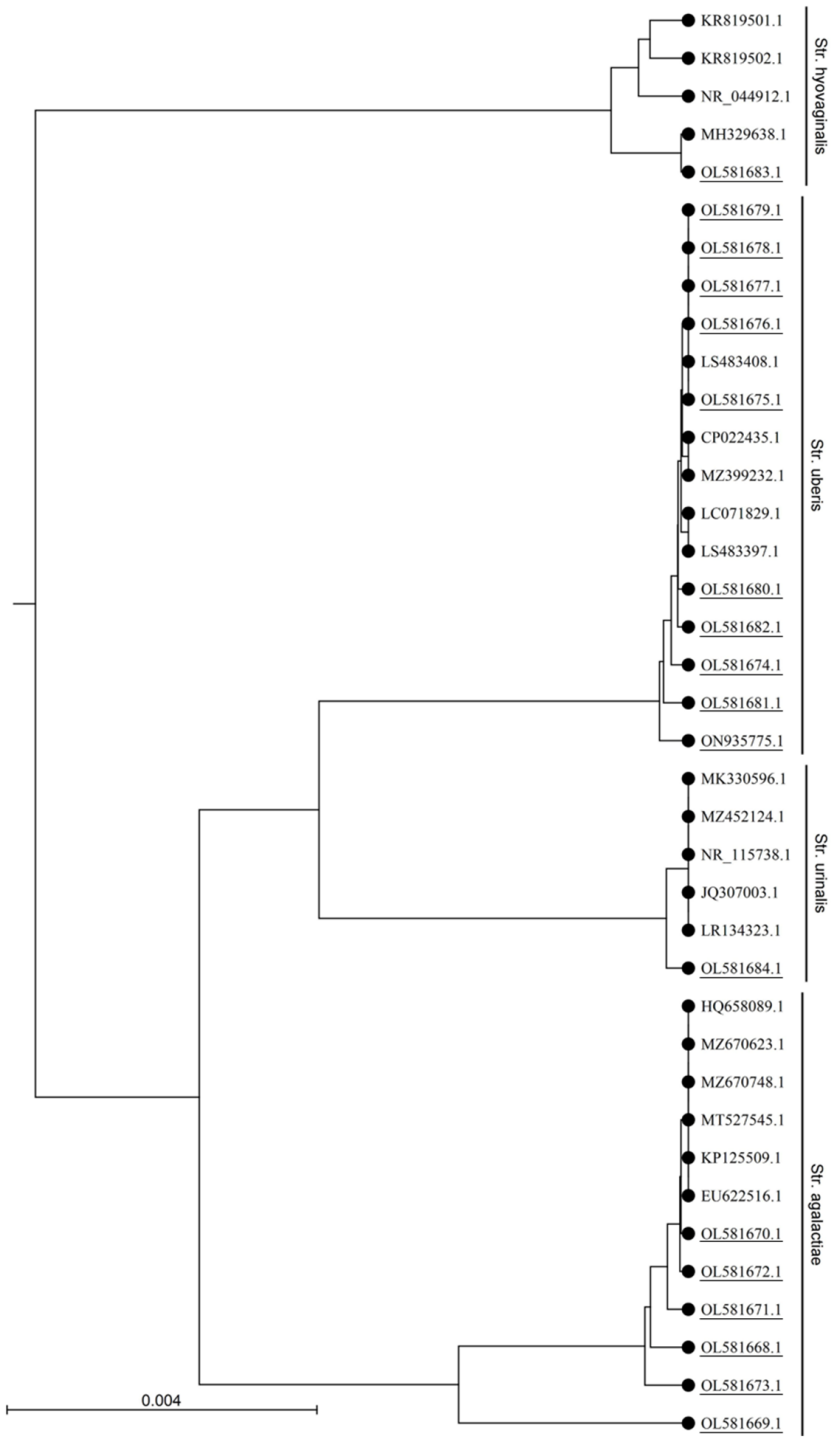
Phylogenetic analysis of the *16S rRNA* gene sequences of *Streptococcus* sp. isolated in this study. The UPGMA tree was prepared using CLC sequence viewer 8.0 and the nucleotide distances were calculated using Kimura 80 model. Accession numbers with underlines denotes sequences obtained in this study.

Cultural and PCR based identification revealed 56.2% (59/105), 17.14% (18/105), 9.52% (10/105) and 22.9% (24/105) of the samples (*n* = 105) were positive for *Staphylococcus* spp., *Streptococcus* spp., *E. faecalis* and *E. coli*, respectively. *Streptococcus* spp. was detected in 18 of 80 (22.5%) milk samples examined from CCM (Table 1). However, none of the samples from apparently healthy cattle carried *Streptococcus* spp. Out of 18 *Streptococcus* positive cattle 5 (27.8%) carried *E. coli,* 6 (33.3%) carried *Staphylococcus*, 1 (5.6%) carried both *E. coli* and *Staphylococcus* (5.6%), indicated that 66.67% *Streptococcus* positive cattle carried mixed infection. *Streptococcus* spp. were sequenced targeting *16S rRNA* and identified as *S. agalactiae* (33.3%), *S. uberis* (55.6%), *S. hyovaginalis* (5.6%), and *S. urinalis* (5.6%) (Figure 1) through homology search, and phylogenetic analysis (Accession no. OL581668 to OL581684, and ON935775). *S. uberis* was most prevalent followed by *S. agalactiae* (Table 1). None of the samples carried *S. dysagalactiae*.

**Table 1:** Frequency of isolation and antibiotic resistance pattern of the *Streptococcus* spp. isolated in this study.

Species	Strain	Sample positive (%) (*n* = 18)	Antibiotic sensitivity pattern (no. of antimicrobial classes)	MDR
*S. agalactiae*	2010	33.33	AZM-CAZ-CIP-CTR-GEN-K-P-S (5)	55.6%
	2013		CIP-K-P (3)	
	2014		CAZ-CIP-CN-CTR-GEN-K-P-S-TET (5)	
	2087		CAZ-CIP-CTR-GEN-K-P-S-TET (5)	
	2090		AMP-AMX-CIP-GEN-P-TET (5)	
	2095		AMP-AMX-AZM-CAZ-CTR-P-TET (6)	
*S. uberis*	2002	55.56	AZM-CIP-CN-K-P-TET (6)	
	2021		CIP-CN-CTR-S-TET (4)	
	2053		CAZ-CIP-CTR-S (3)	
	2055		CIP-TET (2)	
	2058		CIP-TET (2)	
	2060		P-TET (2)	
	2062		CIP-TET (2)	
	2063		CIP-TET (2)	
	2065		CIP-TET (2)	
	2066		CIP-TET (2)	
*S. hyovaginalis*	2022	5.56	CAZ-CIP-CN-CTR-GEN-K-P-S-TET (5)	
*S. urinalis*	2042	5.56	–	

AZM, azithromycin; AMP, ampicillin; AMX, amoxicillin; CAZ, ceftazidime; CIP, ciprofloxacin; CN, cephalexin; CTR, ceftriaxone; K, kanamycin; GEN, gentamicin; P, penicillin; S, streptomycin; TET, tetracycline.

### Antibiotic susceptibility of *Streptococcus* isolates

Fifty-five percent (55.6%, 10/18) of the *Streptococcus* isolated in this study showed multidrug resistance (MDR), with 100% of the *S. agalactiae* and *S. hyovaginalis*. Whereas, *S. urinalis* was susceptible to all the antimicrobials evaluated in this study (Table 1). On the other hand, only 30% of the *S. uberis* showed MDR, and they were susceptible to most of the antibiotics used in this study (Table 1). Among the antibiotics tested, the highest resistance was encountered against ciprofloxacin (83.3%) followed by tetracycline (77.8%). All (100%) of the *Streptococcus* isolates were sensitive to chloramphenicol and vancomycin (Table 1).

### General features of the *Streptococcus uberis* genomes

**Figure 2:**
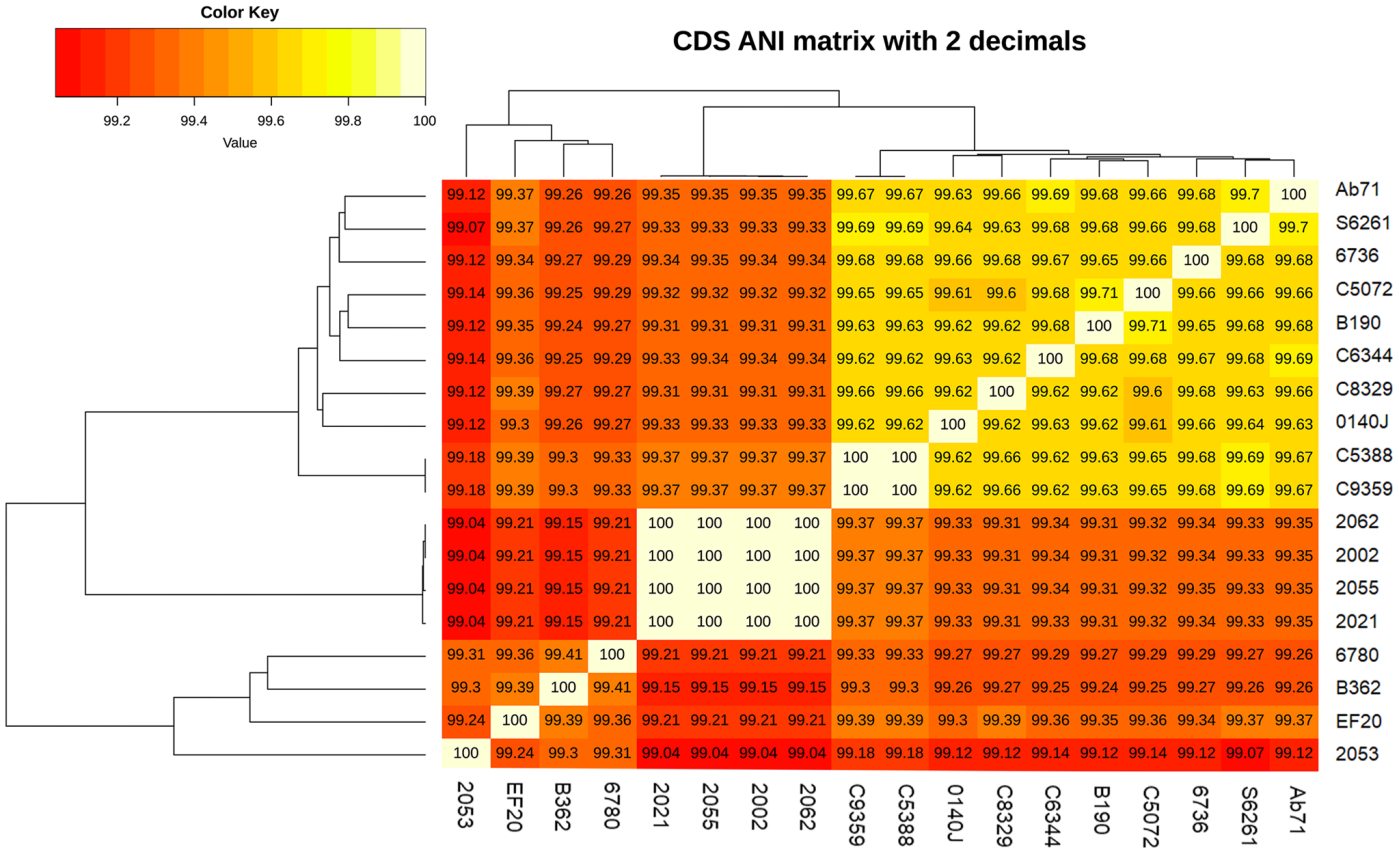
Average nucleotide identity (ANI) of *Streptococcus uberis* genomes described in this study and related strains from UK (34). The nucleotide identity was calculated with get_homologue.pl.

**Figure 3:**
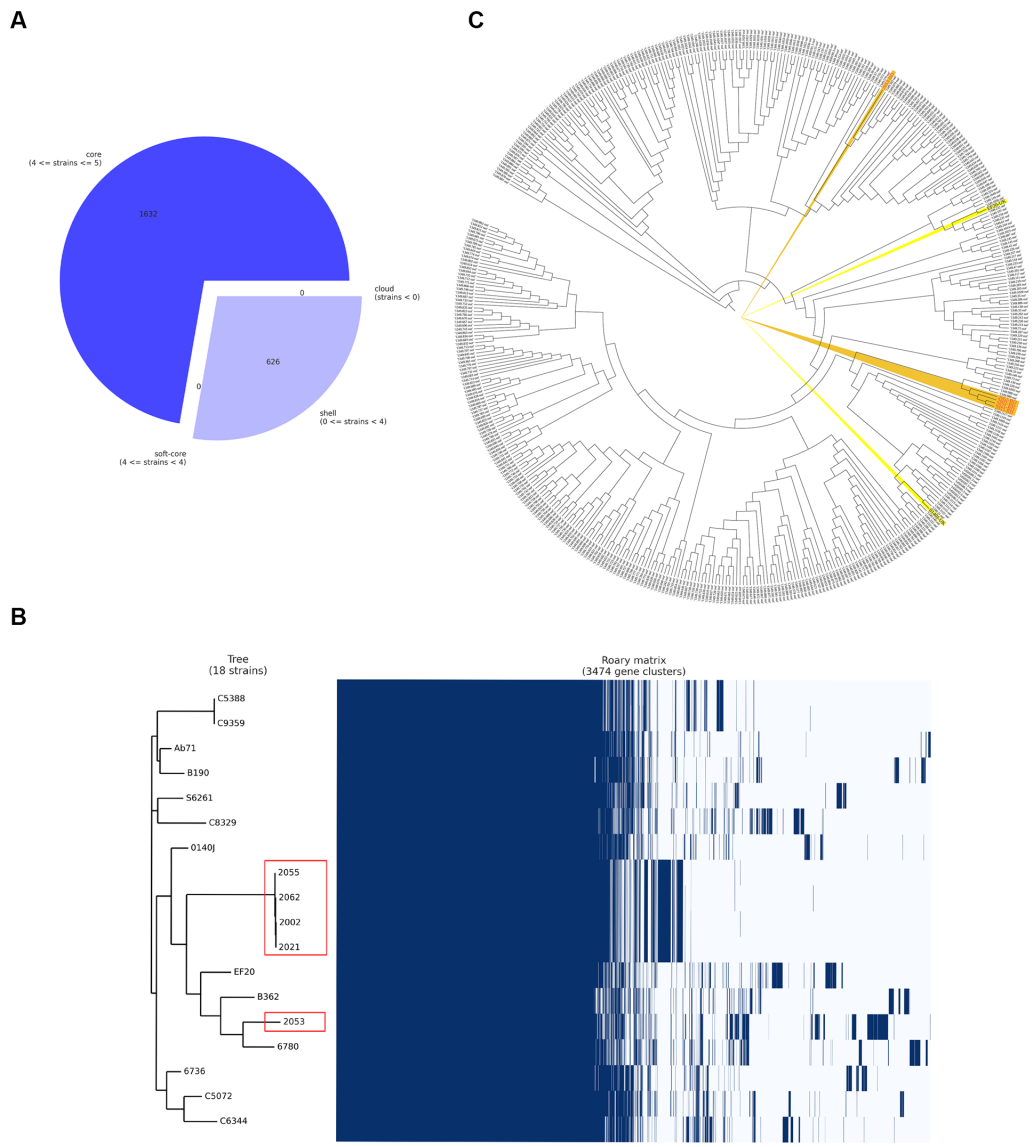
Pangenome analysis of the *S. uberis* isolated from Bangladesh (BD). **(A)** Breakdown of genes in *S. uberis* isolates from BD, **(B)** pangenome based (gene presence and absence) gene clustering matrix of BD (enclosed in red boxes) and UK isolates and **(C)** core genome-based phylogeny with *S. uberis* genomes described throughout the world. The figures were prepared based on the data acquired from Roary pangenome analysis using roary_plots.py script.

Five *S. uberis* isolated in this study were sequenced and assembled to elucidate their genomic features. The assembled genome length ranged from 18,86,469 to 20,33,655 bp with GC contents of 36.4% to 36.5% (Table 2). The raw sequences and draft genomes were submitted to NCBI-bioproject (Accession numbers: PRJNA856652, PRJNA856654, PRJNA856664, PRJNA856665, PRJNA856669), GenBank (Accession numbers: JANLBK000000000–JANLBO000000000) and sequence read archive (SRA) (SRR21812789, SRR21813362, SRR21813574, SRR21813756, SRR21814019). The draft genome of strain 2053 was the largest and strain 2055 being the smallest among the genomes examined in this study. Strains 2002, 2021, 2055 and 2062 carried 100% identical nucleotide sequences, but 2053 shared 99.04% sequence identity with them (Figure 2). The isolates carried 1632 core genes and 626 shell genes (Figure 3A). Gene clustering based on the presence and absence of genes in *S. uberis* revealed identical patterns of the genome 2002, 2021, 2055 and 2062 on a pangenome matrix (Figure 3B) and clustered close to the pathogenic strain 0140J, whereas the strain 2053 clustered close to the non-pathogenic strain EF20 described from the UK (34) (Figure 3B). Similar pattern of clustering was evident on a phylogenetic tree constructed with core genome sequences of *S. uberis* genomes described throughout the world (Figure 3C).

**Table 2:** The genomic features of five *Streptococcus uberis* strains isolated from clinical mastitis milk of dairy cows.

Genome features	2002	2021	2053	2055	2062
Genome size (bp)	1,893,816	1,893,608	2,033,655	1,886,469	1,894,531
Genome coverage (*x*)	30	30	30	30	30
Total contigs	28	25	33	21	19
GC (%)	36.4	36.4	36.5	36.4	36.4
CDS	1848	1847	2032	1840	1845
N50	235,908	235,908	442,384	235,908	419,167
Sequence type	Unknown	Unknown	Unknown	Unknown	Unknown
AMR gene	*tetL, tetM, mreA, patB, lnuD, qac*	*tetL, tetM, mreA, patB, lnuD, qac*	*mreA, patB, qac*	*tetL, tetM, mreA, patB, lnuD, qac*	*tetL, tetM, mreA, patB, lnuD, qac*
Virulent gene	40	40	36	40	40
Number of subsystems	222	222	228	222	223
Subsystem coverage (%)	31	31	29	31	31

GC, guanine-cytosine content; CDS, protein coding sequence; N50, sequence length of the shortest contig (bp) at 50% of the total assembly length.

**Figure 4:**
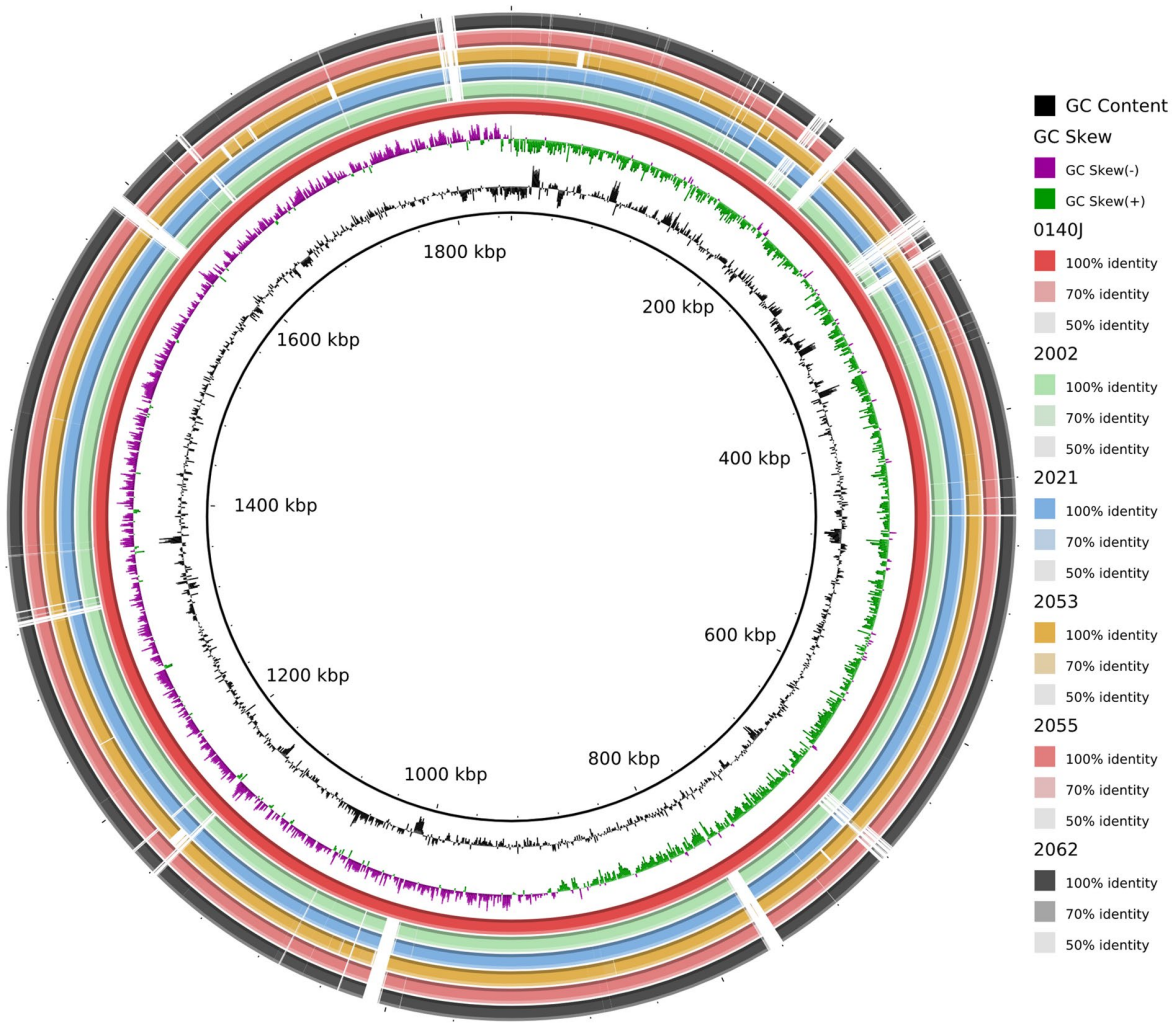
Circular representation of the *S. uberis* complete genomes. Circles (from inside to outside) 1 and 2 (GC content; black line and GC skew; magenta and green lines), circle 3 (*S. uberis* strain 0140J; maroon circle); circle 4 (mapped *S. uberis* strain 2002 genome with *S. uberis* strain 0140J genome; light green circle); circle 5 (mapped *S. uberis* strain 2021 genome with *S. uberis* strain 0140J genome; deep sky circle); circle 6 (mapped *S. uberis* strain 2053 genome with *S. uberis* strain 0140J genome; orange circle); circle 7 (mapped *S. uberis* strain 2055 genome with *S. uberis* strain 0140J genome; brown circle), and circle 8 (mapped *S. uberis* strain 2062 genome with *S. uberis* strain 0140J genome; black circle). BRIG 0.95 was used to build the circular representation. Mapping studies were done using BLASTn with an *e*-value cut-off 1*e*-5.

A comparative genomic analysis was performed with the study strains and the pathogenic close relative *S. uberis* strain 0140J genome (GenBank accession no.: AM946015.1). The circular genome map generated by the BRIG shows that five *S. uberis* strains share high similarities among themselves. In addition, the genomic map obtained from the BRIG comparison revealed that multiple regions were absent in the study genomes in comparison to reference genome and vice versa (Figure 4), suggesting the possible acquisition and/or loss of several additional genes and recombination which may have evolutionarily favored their competitiveness in mastitis pathogenesis. Moreover, some of the predicted pathogenicity and resistance islands overlap with part of those regions (Figure 4).

**Figure 5:**
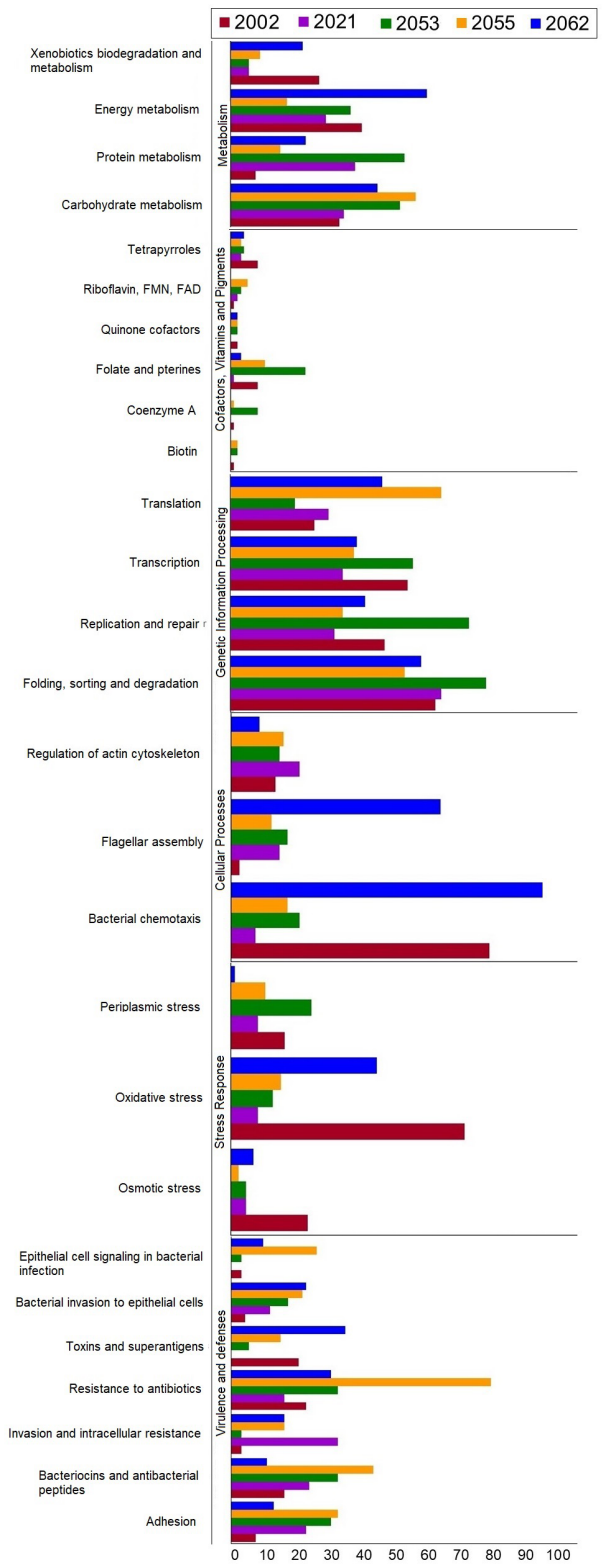
The number of genes categorized by KEGG functional pathways and SEED subsystems annotation of *S. uberis* strains 2002, 2021, 2053, 2055 and 2062.

The coding sequences (CDSs) of the five *S. uberis* strains were annotated using the KEGG pathways and SEED subsystems which classified CDSs into six subcategories including metabolism, cofactors, vitamins and pigments, genetic information processing, cellular processing, stress response and virulence and defenses (Figure 5). The strains 2002, 2021, 2053, 2055 and 2062 were found to conserve 1848, 1847, 2032, 1840 and 1845 CDSs, respectively, that were further divided into 27 functional KEGG subcategories and subsystems according to the aforementioned six categories (Figure 5; Supplementary Table S4). Notably, all of the strains contained a significantly higher number of genes related to genetic information processing (1,149 CDSs), followed by metabolism (883 CDSs), virulence and defenses (531 CDSs), cellular processing (346 CDSs), stress response (243 CDSs) and cofactors, vitamins and pigments (100 CDSs) metabolism (Figure 5; Supplementary Table S4). On an average, *S. uberis* strain 2062 harbors higher number of CDSs (*n* = 767) for KEGG subcategories and SEED subsystems followed by *S. uberis* strain 2053 (*n* = 733), *S. uberis* strain 2001 (*n* = 682), *S. uberis* strain 2055 (*n* = 663), and *S. uberis* strain 2021 (*n* = 510) (Supplementary Table S4).

### Sequence type of the *Streptococcus uberis* isolates

**Figure 6:**
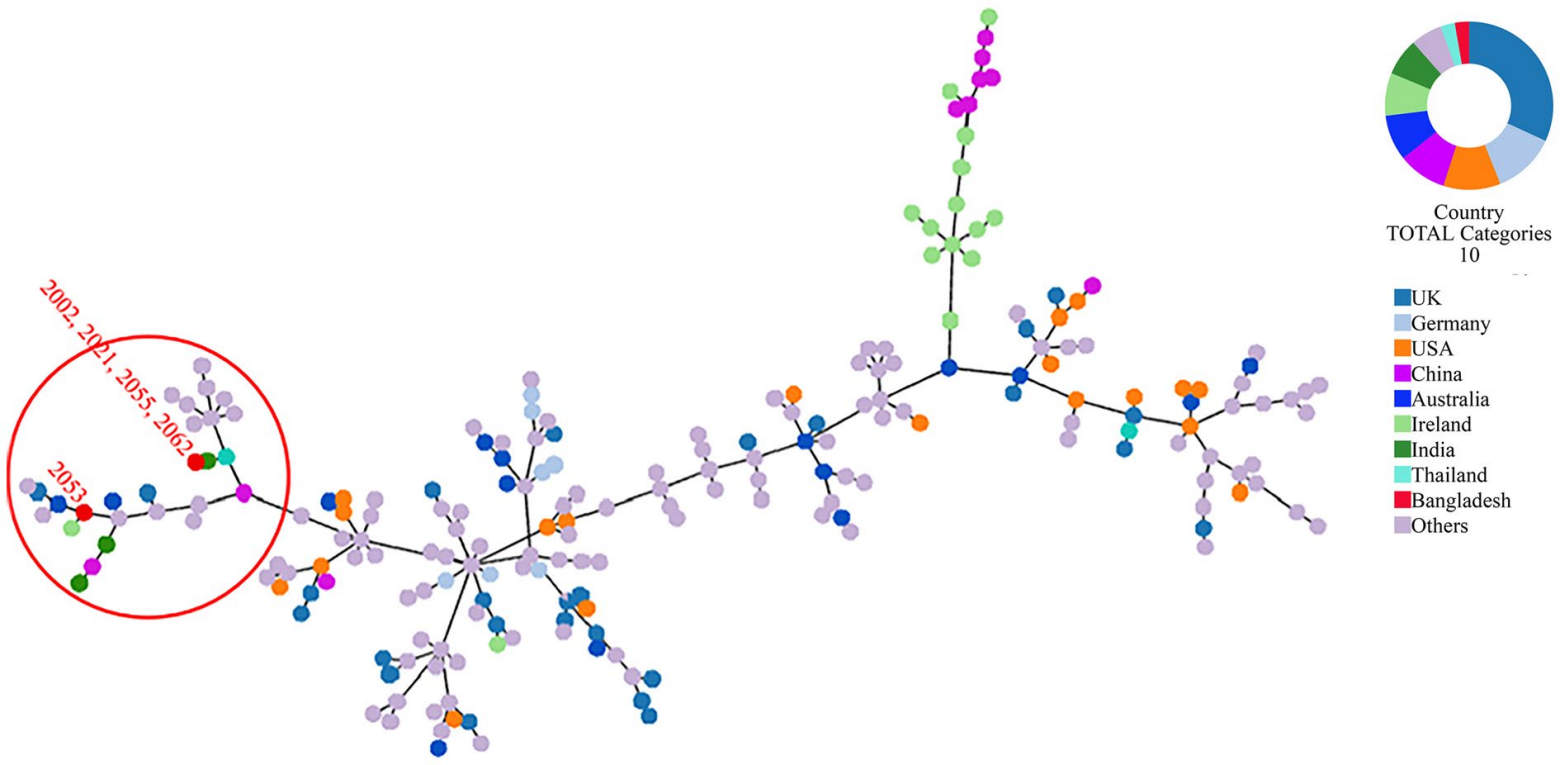
MLST phylogeny of *S. uberis* sequences obtained in this study. Concatenated ST sequences of *S. uberis* were downloaded from PubMLST (https://pubmlst.org/organisms/streptococcus-uberis) and the minimum spanning tree was constructed using goeBurst algorithm of Phyloviz (PHYLOViZ Online). Red dot inside the circle denotes isolates described in this study from Bangladesh.

Whole genome-based sequence typing through the PubMLST website revealed the existence of two novel ST (unknown according to PubMLST) sequences in the *S. uberis* isolated from Bangladesh (BD). One ST is close to ST474 (isolates 2002, 2021, 2055, and 2063), and the other (2053) was close to several ST sequences of ST152, ST133, ST82 and ST968. Phylogeny of the concatenated ST sequences clustered the former group with sequences described from India, Thailand and China. On the other hand, ST sequences of 2053 closely clustered with isolates from Ireland, UK and Australia (Figure 6).

### Virulence factor and antibiotic resistance in the *Streptococcus uberis* isolates

**Figure 7:**
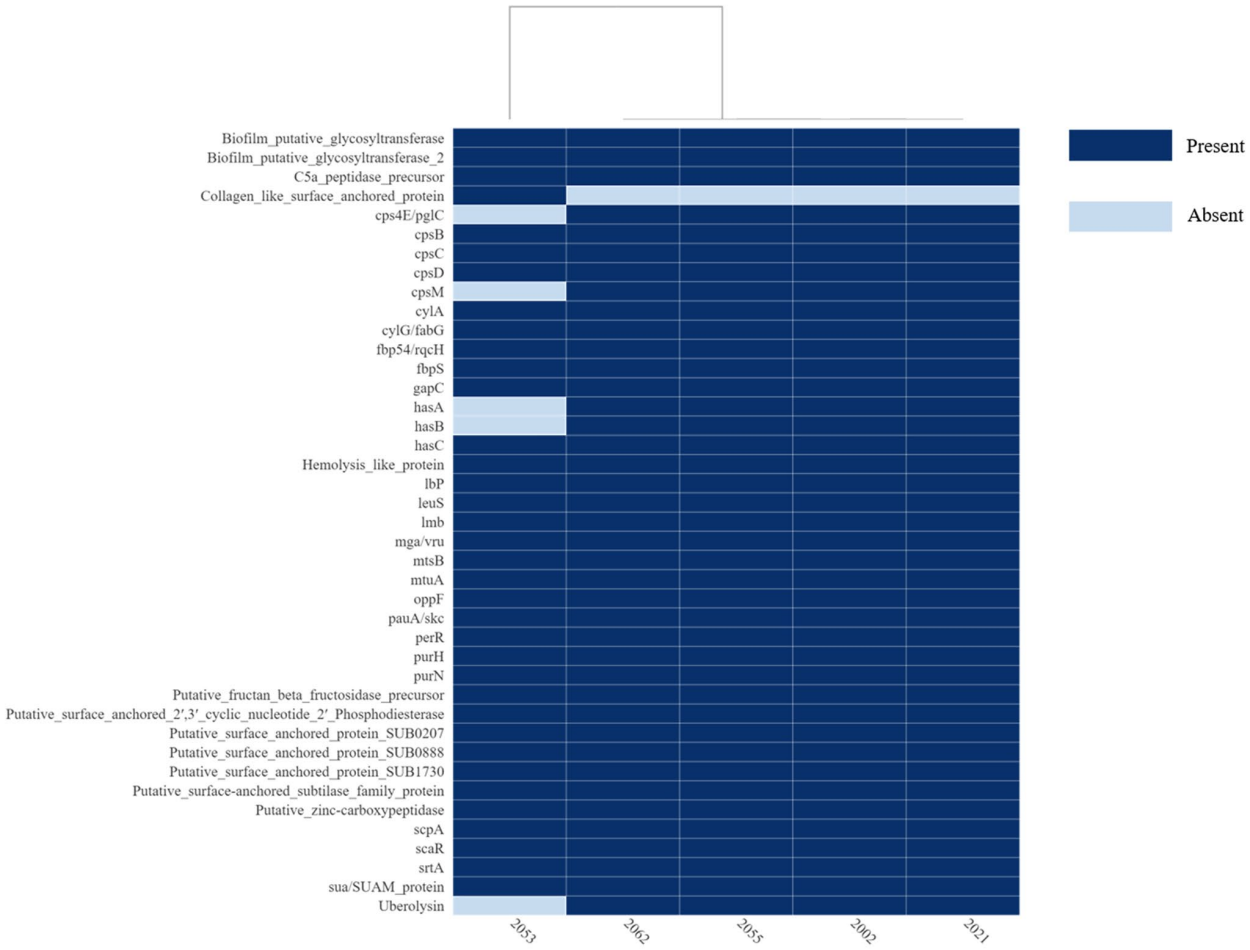
Heatmap of the virulence or putative virulence genes/factors present or absent in the *S. uberis* genomes from Bangladesh. The isolate ID is in the *x*-axis and the virulence genes/factors are in the *y*-axis. The figure was prepared on DISPLAYR (https://southeastasia.displayr.com/) using default parameters and dendrogram appearance.

**Figure 8:**
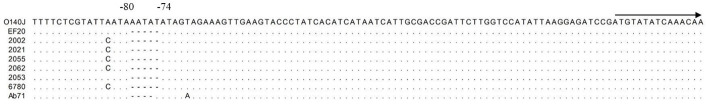
Analysis of the upstream of *vru* gene homologues in the *S. uberis* isolated in this study. Upstream of the *vru* was identified according to the criteria described (34) and the alignment was performed on CLC Genomic Workbench 22.0. The initiation of the *vru* homologue is indicated with arrow. Matching residues were replaced with dots and the deleted residues are marked with dashes. Deletion of 5 pb TATAA was observed in all the strain (2002, 2021, 2053, 2055, 2062) described in this study.

The *S. uberis* genomes were examined for the presence of sixty-one (61) putative virulence genes described so far. The virulence factors and corresponding orthologs identified in the *S. uberis* genomes are provided in Figure 7 and Supplementary Table S5. All the isolates recovered in this study carried thirty-five (35) of the virulence genes examined. In addition, strains 2002, 2021, 2055 and 2062 carried five virulence genes such as *cps4E/pglC, cpsM, hasA*, *hasB,* and uberolysin encoding genes which were absent in strain 2053. On the other hand, 2053 carried one putative virulence factor, “collagen-like surface-anchored protein,” which was absent in the 4 other isolates. Analysis of the *has* operon revealed that all four positive isolates carried *hasA* and *hasB* gene in chronicles, but the *hasC* gene was located 3.9 kb downstream of the *hasAB* and in the opposite direction. In addition, upstream of the VMHJH_06810, VMHJH1_07130, VMHJH2_06655, BAUJH3_05795, BAUJH4_06250 locus of the sequences described in this study, which were homologous to *vru* (SUB0144) of strain 0140J was analyzed for a four bp deletion region as described earlier (34). Unlikely 0140J, a five bp (TATAA) deletion was observed in the position-75 to-79 in all the *S. uberis* isolates described in this study (Figure 8).

Analysis of the *S. uberis* genome through CARD revealed the presence of *tetL, tetM, patA, patB* and *lnuD* genes in all but in strain 2053 only the *patA* and *patB* genes were evident. The genes *tetL* and *tetM* confer resistance to tetracyclines, *patA* and *patB* to fluoroquinolones and *lnuD* to lincosamide antibiotics. The antibiotic resistance genotype correlates with the phenotypic ciprofloxacin and tetracycline resistance pattern of the isolates described in this study (Table 1).

### Analysis of the CRISPR-Cas regions

**Figure 9:**
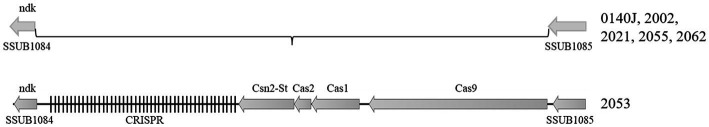
Type-II CRISPR region in the *S. uberis* isolates. Figure showing the relative position of the CRISPR-Cas genes in the *S. uberis* genome. *S. uberis* strain 0140J was used as the reference genome. The CRISPR-Cas system was detected only in strain 2053 among the five *S. uberis* isolates sequenced in this study.

*Streptococcus uberis* isolated in this study were examined for CRISPR-Cas regions where the region was found deleted in all the strains except 2053 when compared to that with reference strain 0140J. In strain 2053 a Type II CRISPR/Cas system was identified comprising *cas9*_*cas1*_*cas2*_*csn2-st* genetic arrangement (Figure 9).

### Prophage and other mobile genetic elements

Prophages are significant in the evolution of bacteria and their virulence. The *S. uberis* genomes were examined for the presence of prophages through the PHASTER website. All the isolates carried two to four prophages; however, intact prophages were detected only in strain 2053 (Table 3). The strain 2053 carried two intact prophages of 44.8 kb (locus_tag: VMHJH2_06750–07050) and 33.7 kb (locus_tag: VMHJH2_09380–09605) sizes with 39.17% and 37.43% GC contents, respectively. Prophages of 8.6 to 11Kb sizes were present in the other 4 isolates, but they were incomplete (Table 3). In further analysis, the intact phages contained fifty-seven and forty-four protein sequences, respectively, where most of the proteins were phage-related (forty-seven and thirty-five sequences, respectively). No known virulence related genes were found in the prophage sequences. The *S. uberis* strains did not carry any plasmids but carried a number of insertion sequences (Table 4). None of the insertion sequences was also associated with virulence determinants.

**Table 3:** Distribution of prophages in the *S. uberis* isolated in this study.

Strain	Region	Length	Completeness	CDS	Possible phage	GC%
2002	1	8.6 Kb	Incomplete	11	PHAGE_Strept_9874_NC_031023	37.88
	2	11 Kb	Incomplete	10	PHAGE_Clostr_phiCT19406C_NC_029006	31.30
2021	1	8.6 Kb	Incomplete	11	PHAGE_Escher_RCS47_NC_042128	37.88
	2	11 Kb	Incomplete	10	PHAGE_Bacill_CampHawk_NC_022761	31.30
2053	1	26.8 Kb	Incomplete	36	PHAGE_Strept_SpSL1_NC_027396	35.25
	2	20.5 Kb	Incomplete	22	PHAGE_Pectob_phiA41_NC_048659	36.97
	3	44.8 Kb	Intact	57	PHAGE_Strept_A25_NC_02869	39.17
	4	33.7 Kb	Intact	44	PHAGE_Strept_2972_NC_007019	37.43
2055	1	8.6 Kb	Incomplete	11	PHAGE_Cronob_vB_CsaM_GAP32_NC_019401	37.88
	2	11 Kb	Incomplete	10	PHAGE_Rhizob_RR1_A_NC_021560	31.30
2062	1	8.6 Kb	Incomplete	11	PHAGE_Escher_RCS47_NC_042128	37.88
	2	11 Kb	Incomplete	10	PHAGE_Bacill_PfEFR_5_NC_031055	31.30

GC, guanine-cytosine content; CDS, protein coding sequence.

**Table 4:** Transposon and insertion sequences in the *S. uberis* isolated in this study.

IS family	Strain ID (no. of sites)
IS256	2002, 2012, 2055, 2066
IS3	2002 (2), 2021, 2053, 2055, 2062
IS6	2002 (3), 2021 (3), 2055, 2062 (2)
Unknown	2002, 2021 (2), 2053 (2), 2055 (2), 2062 (2)

## Discussion

The present study was aimed to the determination of occurrence of *Streptococcus* spp. and get genetic insight into the *S. uberis* genome isolated from clinical mastitis of cattle in Bangladesh. Milk samples from clinical mastitis (CM) were collected from major dairy farms and a dairy community in Dhaka, Mymensingh and Sirajganj district of Bangladesh. On cultural examination and sequencing of *16S rRNA* gene, milk from 22.5% of the CCM was found positive for *Streptococcus* spp. The frequency of isolation of *Streptococcus* recorded in this study is in consent with the worldwide prevalence of *Streptococcus* spp. in mastitis (4). In this study, four different species of *Streptococcus*, including *S. agalactiae*, *S. uberis*, *S. hyovaginalis* and *S. urinalis* with the highest frequency of *S. uberis* (55.56%) followed by *S. agalactiae* (33.33%) was identified. The increased occurrence of *S. uberis* is in consent with current research throughout the world but differs with that reported from Asia where *S. agalactiae* was predominantly associated with mastitis (4). The difference in the occurrence of *S. uberis* in mastitis is not impossible; however, to reveal the actual scenario and make a generalized statement, country-wide study with more samples is necessary. Besides, we have encountered two new species *S. hyovaginalis* and *S. urinalis* from clinical mastitis for the first time in the world. Although the pathogenicity or association of these two species with mastitis was not studied, they were the sole *Streptococcus* spp. isolated from the respective milk samples, as well as none of the other samples from CCM or apparently healthy cattle carried these two *Streptococcus* spp. indicating their possible association with mastitis in the respective cows; however, as we have used composite milk sample their non-pathogenic presence in the unaffected quarter also cannot be excluded. Moreover, aseptic sample collection from mastitis is pretty critical and enrichment of sample might give advantages for contaminants to be selected if the media does not allow growth of the bacteria that cause clinical mastitis. Thus, further studies on their virulence and possible association with mastitis are under investigation in our laboratory.

Antimicrobial resistance (AMR) is a critical problem making mastitis control challenging through systemic or intramammary therapies. Moreover, extensive use of antimicrobials in mastitis control resulted in antimicrobial residue in milk and entering the human body through the food chain (41). In this study, we found that 100% of the *S. agalactiae* and *S. hyovaginalis* were MDR, whereas antibiotic resistance was observed in 30% of the *S. uberis* and none of the *S. urinalis*. The highest resistance was observed against ciprofloxacin and tetracycline (83.3%), which correlates with the antibiotic uses in the study farms described in our previous study (18) as well as follows the global trends of antibiotic resistance in *Streptococcus* spp. (42). Interestingly, *S. uberis* was sensitive to most of the antibiotics tested in this study with 100% sensitivity to ampicillin, amoxicillin, bacitracin, chloramphenicol, gentamicin, vancomycin; 90% to ceftazidime and 80% to penicillin. On the other hand, *S. agalactiae* showed resistance to penicillin (100%), ceftazidime and gentamicin (66.7%), ampicillin and amoxicillin (33.3%). Increased resistance in *S. agalactiae* is not unusual, and their increased resistance in comparison to *S. uberis* and *S. dysagalactiae* has been reported earlier (4). The overall findings represents a variable resistance pattern of *Streptococcus* spp., warning for an antibiotic susceptibility testing before prescribing any antibiotic to treat streptococcal mastitis to ensure prudent use and prevent subsequent threat of transferring antibiotic residue to human through milk.

In this study, the prevalence of *S. uberis* was documented highest (55.6%). Considering the worldwide increasing trend of *S. uberis* in bovine mastitis (4, 13), this study was further extended to characterize the *S. uberis* isolates through WGS. Sequencing of the five representative isolates followed by assembly revealed the genome sizes ranging from 1,886,469 bp (strain 2055) to 2,033,655 bp (strain 2053) with 36.4 to 36.5% GC contents, respectively. The genome sizes correspond to the *S. uberis* genomes reported earlier (33, 34). The variation in genome was investigated and annotation revealed the presence of phages and insertion sequence of variable lengths. The genome of strain 2053 carried two complete phages and a total of around 140 kb phage-related sequences that were absent in the other four genomes, which might have greatly contributed to its increased genome size. Pan-genome analysis, whole genome phylogeny and MLST revealed the presence of two unknown sequence types (STs) of *S. uberis* in the study populations. Similarly, novel ST types have been reported earlier from different countries (4, 33, 34, 43), which might be related to the limited databases of *S. uberis* genomes. The clustering of four out of five isolates with India, Thailand and China indicated the circulation of similar STs in this subcontinent. Moreover, the *S. uberis* strains isolated and sequenced in this study was found to sharing more common regions of genetic variation with the reference genomes 0140J at different sites on the genome. The great genomic resemblance between the study strains and the *S. uberis* strain 0140J, another mastitis clone from the European continent indicates the possible common ancestry between these lineages, despite the wide geographical distance where the strains were originated. However, with the few isolates analyzed in this study an overall diversity of *Streptococcus* could not be completely described; thus, further country wide studies with more samples and isolates are recommended to elucidate the genomic diversity of *S. uberis* circulating in Bangladesh as well as predominance of certain STs in this sub-continent.

*Streptococcus uberis* is known to establish a successful intramammary infection through the exploitation of different virulence factors associated with invasion of mammary epithelium, evasion of host immunity, binding antimicrobial compounds, biofilm formation through the acquisition of amino acids by breaking milk protein casein (4). Although the virulence determinant in *S. uberis* is not completely understood; several putative virulence determinants have been described previously (4, 33, 34, 43). Considering the previous reports and *S. uberis* putative virulence database (suPVDB), a total of sixty-one (61) putative virulence factors were investigated, where forty-one (41) virulence factors were identified in the studied genomes. Among the virulence factors thirty-five virulence factors were common to all, including genes encoding for major virulence factors relating to the adhesion to epithelial cells (*srtA, fbp54*/*rqcH* and *fbps*), colonization (*lmb*), invasion/penetration and dissemination (*pauA*/*skc*), immuno invasion through the synthesis of hyalurinic acid capsule (*hasC*) and delayed accumulation of lymphocytes (*scpA* and C5a peptidase), biofilm formation (*cpsBCD*), hemolytic activity (*cylAG* and hemolysis like protein) and virulence regulator (*mga*/*vru*) which is known to regulate a number of phenotypes such as growth, biofilm formation and resistance to phagocytosis (33, 44, 45).

In addition, five (*cps4E/pglC*, *cpsM*, *hasA*, *hasB* and uberolysin encoding gene) were native to the strains 2002, 20,021, 2055 and 2066. HasABC is associated with the formation of the hyaluronic acid capsule, which was required for the prevention of opsonization and phagocytosis as well as escaping neutrophil traps (46); however, some authors claimed they are non-essential or play a minor role in the induction of mastitis by *S. uberis* (33, 47, 48). Similarly, bacteriocin provides competitive benefits to the bacterium for surviving in a complex environment by inhibiting the growth of other bacteria. The genome 2053 carried five different genes associated with bacteriocin production, similar to the other genome in this study except for uberolysin. The absence of uberolysin might not render strain 2053 avirulent but may give additional benefits or fitness to the other isolates carrying the gene; however, further investigations are necessary to establish the associations.

The *vru* gene was reported to regulate a number of virulence genes, and inactivation of this gene was responsible for reduced mammary gland colonization and clinical signs (49). A four bp deletion at the upstream (−76 to −79) was speculated to play an important role in the regulatory function of *vru* gene and subsequently influence host-pathogen interactions (34). In this study, all five genomes had a five bp deletion (TATAA) at −75 to −79 positions similar to that reported in EF20, a non-virulent *S. uberis* strain reported from the UK (34). Considering the putative virulence gene makeup, although strain 2053 closely clustered with EF20, they are genetically different, and the deletion at upstream of the *vru* gene might not be critical to the virulence of the isolates. However, as we did not perform phenotypic studies, making any concrete statement from our findings might be misleading. Thus, further phenotypic experiments with isolates having different variations in the *vru* upstream would provide comprehensive information on its association with the regulation of *vru* gene and virulence of *S. uberis* in bovine mastitis.

We further sought to determine potential metabolic functions of the five *S. uberis* strains described in this study. The KEGG pathways and SEED subsystems analysis uncovered significant differences in metabolic functions among the study strains. All KEGG and SEED subsystem characterized proteins in five strains were assigned into six different categories representing 27 subcategories. Of the identified subcategories, genes associated with the carbohydrate, protein and energy metabolism, translation, transcription, bacterial chemotaxis, flagellar assembly and oxidative stress remained highest in the study genomes, which might be associated with host-pathogen interactions during the progression of mastitis. These findings corroborated with many of the earlier studies on bovine mastitis pathogens (50–52). Further work is needed to verify these findings and metabolic gene reservoirs and the mechanism of transfer across different strains and/or with their hosts.

## Conclusion

Genomic information of *S. uberis* generated in this study revealed two novel STs and possible virulence makeup that will significantly contribute to the development of new tools for virulence profiling and ST typing of *S. uberis*. The sequence data generated in this study could also be used for the exploration of the genomic background behind the evolution of this pathogen and the story behind its enhanced environmental fitness over time.

## Data availability statement

The datasets presented in this study can be found in online repositories. The names of the repository/repositories and accession number(s) can be found in the article/Supplementary material.

## Ethics statement

The animal study was reviewed and approved by Animal Welfare and Experimentation Ethics Committee (AWEEC), Bangladesh Agricultural University, Mymensingh-2,202, Bangladesh Approval No. AWEEC/BAU/2020(45). Written informed consent was obtained from the owners for the participation of their animals in this study.

## Author contributions

JH and MSK received the project funds, planned research and manage laboratory activities. Lab work was conducted by JH, MAB, and MWA. Analysis of genomic data and curation was performed by JH, MMH, MNH, and AK. The manuscript was drafted and revised by JH, AK, MTR, MSI, and MNH. All authors contributed to the article and approved the submitted version.

## Funding

This research was funded by Bangladesh Agricultural University Research System (BAURES) (Project no. 2021/21/BAU).

## Conflict of interest

The authors declare that the research was conducted in the absence of any commercial or financial relationships that could be construed as a potential conflict of interest.

## Publisher’s note

All claims expressed in this article are solely those of the authors and do not necessarily represent those of their affiliated organizations, or those of the publisher, the editors and the reviewers. Any product that may be evaluated in this article, or claim that may be made by its manufacturer, is not guaranteed or endorsed by the publisher.

## Footnotes

^1^ https://en.banglapedia.org/index.php/Dairy_Farm

^2^ https://rast.nmpdr.org/rast.cgi

^3^ https://card.mcmaster.ca/

^4^ www.mgc.ac.cn/VFs

^5^ https://Proksee.ca

^6^ https://phaster.ca

^7^ https://pubmlst.org

^8^ https://pubmlst.org/organisms/streptococcus-uberis

^9^ PHYLOViZ Online
