# Supplementary material for: Diversity of Streptococcus spp. and genomic characteristics of Streptococcus uberis isolated from clinical mastitis of cattle in Bangladesh
Source: Front Vet Sci. 2023 Jul 18;10:1198393. doi: 10.3389/fvets.2023.1198393 (PMC10392839; doi:10.3389/fvets.2023.1198393)
Supplement: Supplementary file 1 [file Table_1.docx]

**Diversity of *Streptococcus* spp. and genomic characteristics of *Streptococcus uberis* isolated from clinical mastitis of cattle in Bangladesh**

Jayedul Hassan, Md. Abdus Sattar Bag, Ajran Kabir, Md. Wohab Ali, Md. Nazmul Hoque, Maqsud Hossain, Md. Tanvir Rahman, Md. Shafiqul Islam, Md. Shahidur Rahman Khan

**Supplementary Table 1.** Characteristics of the dairy farms/ community included in this study

| Dairy units (no. of farms) | | No. of Dairy Cattle | Lactating cattle | Cattle with mastitis | Frequency of mastitis |
| --- | --- | --- | --- | --- | --- |
| A | | 1535 | 450 | 14 | 3.11% |
| B | | 174 | 60 | 11 | 18.33% |
| C | | 225 | 75 | 16 | 21.33% |
| D | | 600 | 160 | 12 | 7.50% |
| DCS (15) | 1 | 18 | 6 | 02 | 27.55% |
|  | 2 | 20 | 8 | 03 |  |
|  | 3 | 05 | 2 | 01 |  |
|  | 4 | 16 | 9 | 02 |  |
|  | 5 | 05 | 2 | 01 |  |
|  | 6 | 20 | 11 | 01 |  |
|  | 7 | 22 | 13 | 03 |  |
|  | 8 | 16 | 09 | 01 |  |
|  | 9 | 11 | 05 | 02 |  |
|  | 10 | 06 | 03 | 01 |  |
|  | 11 | 20 | 11 | 03 |  |
|  | 12 | 05 | 03 | 01 |  |
|  | 13 | 14 | 08 | 02 |  |
|  | 14 | 07 | 03 | 02 |  |
|  | 15 | 12 | 05 | 02 |  |
|  | Sub-total | 197 | 98 | 27 |  |
| Total | | 2731 | 843 | 80 | 9.48% |

Legend: A-D: four government dairy farms located at Dhaka and Mymensingh districts; DCS, dairy community at Shahjadpur, Sirajgonj district of Bangladesh.

**Supplementary Table 2.** Primer used for the identification of *Streptococcus* spp. by PCR

| **Name of the primer** | **Sequence (5’-3’)** | **Target species** | **PCR conditions** | **Reference** |
| --- | --- | --- | --- | --- |
| NUCSEQ1 | ACAGGCGTTTTAATCCTTGC | *Staphylococcus spp.* | 30× (95°C 30 s, 55°C 30 s, 72°C 30 s) | Baron et al., 2004 |
| NUCSEQ2 | TCCTTTTGGGCTTGTTCAAT |  |  |  |
| F1 | GAGTTTGATCATGGCTCAG | *S. agalactiae* | 10× (94°C 30 s, 55°C 30 s, 72°C 30 s)  22× (94°C 30 s, 51°C 30 s, 72°C 30 s) | Martinez et al., 2001 |
| IMOD | ACCAACATGTGTTAATTACTC |  |  |  |
| lytR-F | ATGAAAATTGGAAAAAAAATA | *S. dysagalactiae* | 35× (95°C 30 s, 42°C 30 s, 72°C 30 s) | Alves-Barroco et.al., 2019 |
| lytR-R | TTAAGGAAGAGAGGTGGTTGTA |  |  |  |
| STRU-UbI | TAAGGAACACGTTGGTTAAG | *S. uberis* | 30× (94°C 30 s, 55°C 30 s, 72°C 30 s) | Hassan et al., 2001 |
| STRU-UbI | TCCAGTCCTTAGACCTTCT |  |  |  |
| ddl*_E. faecalis_*-F | ATCAAGTACAGTTAGTCTTTA | *E. faecalis* | 30× (95°C 30 s, 48°C 30 s, 72°C 60 s) | Dutka-Malen et al., 1995 |
| ddl*_E. faecalis_*-R | AACGATTCAAAGCTAACT |  |  |  |
| ddl*_E. faecium_*-F | GCAAGGCTTCTTAGAGA | *E. faecium* | 30× (95°C 30 s, 50°C 30 s, 72°C 60 s) |  |
| ddl*_E. faecium_*-R | CATCGTGTAAGCTAACTTC |  |  |  |
| ECO-1 | GACCTCGGTTTAGTTCACAGA | *E. coli* | 30× (94°C 45 s, 58°C 45 s, 72°C 60 s) | Wang et al., 1996 |
| ECO-2 | CACACGCTGACGCTGACCA |  |  |  |
| C2700F | CGWATGAACATYGGBCAGGT | *Corynebacterium* spp. | 30× (94°C 30 s, 60°C 30 s, 72°C 60 s) | Khamis et al., 2004 |
| C3130R | TCCATYTCRCCRAARCGCTG |  |  |  |

Alves-Barroco, C., Roma-Rodrigues, C., Raposo, L. R., Brás, C., Diniz, M., Caço, J., Costa, P. M., Santos-Sanches, I., Fernandes, A. R. (2019). *Streptococcus dysagalactiae* subsp. *dysagalactiae* isolated from milk of the bovine udder as emerging pathogens: In vitro and in vivo infection of human cells and zebrafish as biological models. Microbiologyopen. 8:e00623. doi: 10.1002/mbo3.623.

Baron, F., Cochet, M. F., Pellerin, J. L., BenZakour, N., Lebon, A., Navarro, A., Proudy, I., Le Loir, Y., Gautier, M. (2004). Development of a PCR test to differentiate between *Staphylococcus aureus* and *Staphylococcus intermedius*. J. Food Prot. 67(10):2302-5. doi: 10.4315/0362-028x-67.10.2302.

Dutka-Malen, S., Evers, S., Courvalin, P. (1995). Detection of glycopeptide resistance genotypes and identification to the species level of clinically relevant enterococci by PCR. J. Clin. Microbiol. 33(1):24–27.

Hassan, A. A., Khan, U. I., Abdulmawjood, A., Lammler, C. (2001). Evaluation of PCR methods for rapid identification and differentiation of *Streptococcus uberis* and *Streptococcus parauberis*. J. Clin. Microbiol. 39(4):1618-1621.

Khamis, A., Raoult, D., La Scola, B. (2004). RpoB gene sequencing for identification of Corynebacterium species. *J. Clin. Microbiol*. 42:3925–3931. doi: 10.1128/JCM.42.9.3925-3931.2004

Martinez, G., Harel, J., Gottschalk, M. (2001). Specific detection by PCR of *Streptococcus agalactiae* in milk. Can. J. Vet. Res. 65:68-72.

Wang, R. F., Cao, W. W., Cerniglia, C. E. (1996). PCR detection and quantitation of predominant anaerobic bacteria in human and animal fecal samples. Appl. Environ. Microbiol. 62:1242–1247. https://doi.org/10.1128/aem.62.4.1242- 1247.1996.

**Supplementary Table 3.** Farm wise isolation of *Streptococcus uberis* and their sequence information

| Isolate ID | Farm ID | Geographical location | Accession No. | |
| --- | --- | --- | --- | --- |
|  |  |  | 16S rRNA | Whole genome sequence |
| BAU/MH/Bag-2002 | A | 23.8807 N 90.2765 E | OL581674.1 | JANLBK000000000 |
| BAU/MH/Bag-2021 | B | 23.4357 N 90.2230 E | OL581675.1 | JANLBL000000000 |
| BAU/MH/Bag-2053 | C | 23.8895 N 90.2737 E | ON935775 | JANLBM000000000 |
| BAU/MH/Bag-2055 | D | 24.3343 N 90.2252 E | OL581676.1 | JANLBN000000000 |
| BAU/MH/Bag-2058 |  |  | OL581677.1 |  |
| BAU/MH/Bag-2060 |  |  | OL581678.1 |  |
| BAU/MH/Bag-2062 |  |  | OL581679.1 | JANLBO000000000 |
| BAU/MH/Bag-2063 |  |  | OL581680.1 |  |
| BAU/MH/Bag-2065 |  |  | OL581681.1 |  |
| BAU/MH/Bag-2066 |  |  | OL581682.1 |  |

**Supplementary Table 4.** Distribution of functional KEGG subcategories and subsystems in the studied genomes

| **Categories** | **Subcategories and subsystems** | **2002** | **2021** | **2053** | **2055** | **2062** |
| --- | --- | --- | --- | --- | --- | --- |
| Metabolism | Carbohydrate metabolism | 48 | 50 | 75 | 82 | 65 |
|  | Protein metabolism | 11 | 55 | 77 | 22 | 33 |
|  | Energy metabolism | 58 | 42 | 53 | 25 | 87 |
|  | Xenobiotics biodegradation and metabolism | 39 | 8 | 8 | 13 | 32 |
| Cofactors, Vitamins, Prosthetic Groups, Pigments | Biotin | 1 | 0 | 2 | 2 | 0 |
|  | Coenzyme A | 1 | 0 | 8 | 1 | 0 |
|  | Folate and pterines | 8 | 1 | 22 | 10 | 3 |
|  | Quinone cofactors | 2 | 0 | 2 | 2 | 2 |
|  | Riboflavin, FMN, FAD | 1 | 2 | 3 | 5 | 0 |
|  | Tetrapyrroles | 8 | 3 | 4 | 3 | 4 |
| Genetic Information Processing | Folding, sorting and degradation | 83 | 85 | 112 | 62 | 68 |
|  | Replication and repair | 55 | 37 | 85 | 40 | 48 |
|  | Transcription | 63 | 40 | 65 | 44 | 45 |
|  | Translation | 30 | 35 | 23 | 75 | 54 |
| Cellular Processes | Bacterial chemotaxis | 64 | 6 | 17 | 14 | 95 |
|  | Flagellar assembly | 2 | 12 | 14 | 10 | 52 |
|  | Regulation of actin cytoskeleton | 11 | 17 | 12 | 13 | 7 |
| Stress Response | Osmotic stress | 20 | 4 | 4 | 2 | 6 |
|  | Oxidative stress | 91 | 7 | 11 | 13 | 38 |
|  | Periplasmic Stress | 14 | 7 | 21 | 9 | 1 |
| Virulence and defenses | Adhesion | 7 | 21 | 28 | 30 | 12 |
|  | Bacteriocins and antibacterial peptides | 15 | 22 | 30 | 40 | 10 |
|  | Invasion and intracellular resistance | 3 | 30 | 3 | 15 | 15 |
|  | Resistance to antibiotics | 21 | 15 | 30 | 73 | 28 |
|  | Toxins and superantigens | 19 | 0 | 5 | 14 | 32 |
|  | Bacterial invasion of epithelial cells | 4 | 11 | 16 | 20 | 21 |
|  | Epithelial cell signaling in bacterial infection | 3 | 0 | 3 | 24 | 9 |
|  |  |  |  |  |  |  |
| Total coding sequences/hits | | 682 | 510 | 733 | 663 | 767 |

**Supplementary Table 5.** Distribution of virulence related genes in *Streptococcus uberis* isolated in this study with corresponding locus in the genomes

| Virulence gene/ candidates* | Product based on NCBI PGAP annotation/ blastx | Locus in 0140J | BD isolates with the orthologs | Corresponding locus in BD isolates |
| --- | --- | --- | --- | --- |
| Biofilm_putative_glycosyltransferase | Glycosyltransferase | SUB0538 | 2002, 2021, 2053, 2055, 2062 | VMHJH_05055, VMHJH1_04580, VMHJH2_07685, BAUJH3_04575, BAUJH4_02485 |
| Biofilm_putative_glycosyltransferase_2 | Glycosyltransferase family 4 protein | SUB0539 | 2002, 2021, 2053, 2055, 2062 | VMHJH_05060, VMHJH1_04575, VMHJH2_07680, BAUJH3_04570, BAUJH4_02480 |
| C5a peptidase precursor | S8 family serine peptidase | SUB1154 | 2002, 2021, 2053, 2055, 2062 | VMHJH_05790, VMHJH1_06155, VMHJH2_02730, BAUJH3_07245, BAUJH4_07270 |
| Collagen-like surface-anchored protein | LPXTG cell wall anchor domain-containing protein | SUB1095 | 2053 | VMHJH2_02430 |
| *cps4E/ pglC* | Sugar transferase | SUB1038 | 2002, 2021, 2055, 2062 | VMHJH_06375, VMHJH1_05570, BAUJH3_06665, BAUJH4_06685 |
| *cpsB* | Tyrosine protein phosphatase | SUB1042 | 2002, 2021, 2053, 2055, 2062 | VMHJH_06355, VMHJH1_ 05590, VMHJH2_02135, BAUJH3_06685, BAUJH4_06705 |
| *cpsC* | Wzz/FepE/Etk N terminal domain-containing protein | SUB1041 | 2002, 2021, 2053, 2055, 2062 | VMHJH_06360, VMHJH1_05585, VMHJH2_02130, BAUJH3_06680, BAUJH4_06700 |
| *cpsD* | Tyrosine-protein kinase | SUB1040 | 2002, 2021, 2053, 2055, 2062 | VMHJH_06365, VMHJH1_05580, VMHJH2_02125, BAUJH3_06675, BAUJH4_06695 |
| *cpsM* | Glycosyl transferase | Absent | 2002, 2021, 2055, 2062 | VMHJH_06390, VMHJH1_05555, BAUJH3_06650, BAUJH4_06670 |
| *cylA* | ABC transporter ATP-binding protein/ permease | SUB1689 | 2002, 2021, 2053, 2055, 2062 | VMHJH_03025, VMHJH1_03025, VMHJH2_04740, BAUJH3_03215, BAUJH4_04520 |
| *cylG/ fabG* | 3-oxoacyl reductase | SUB1496 | 2002, 2021, 2053, 2055, 2062 | VMHJH_03965, VMHJH1_03965, VMHJH2_03760, BAUJH3_02275, BAUJH4_03580 |
| *fbp54/ rqcH* | Fibronectin-binding protein | SUB1111 | 2002, 2021, 2053, 2055, 2062 | VMHJH_06010, VMHJH1_05935, VMHJH2_02510, BAUJH3_07025, BAUJH4_07050 |
| *fbpS* | Fibronectin-binding protein | SUB1784 | 2002, 2021, 2053, 2055, 2062 | VMHJH_02535, VMHJH1_02535, VMHJH2_05205, BAUJH3_03705, BAUJH4_05010 |
| *gapC* | Type I glyceraldehyde-3-phosphate dehydrogenase | SUB1630 | 2002, 2021, 2053, 2055, 2062 | VMHJH_03325, VMHJH1_03325, VMHJH2_04470, BAUJH3_2915, BAUJH4_04220 |
| *hasA* | hyaluronan synthase | SUB1697 | 2002, 2021, 2055, 2062 | VMHJH_02985, VMHJH1_02985, BAUJH3_03255, BAUJH4_04560 |
| *hasB* | DUP-glucose 6-dehydrogenase | SUB1696 | 2002, 2021, 2055, 2062 | VMHJH_02990, VMHJH1_02990, BAUJH3_03250, BAUJH4_04555 |
| *hasC* | NAD(P)H-dependent glycerol 3-phosphate dehydrogenase | SUB1691 | 2002, 2021, 2053, 2055, 2062 | VMHJH_03015, VMHJH1_03015, VMHJH2_04750, BAUJH3_03225, BAUJH4_04530 |
| Hemolysis like protein | TlyA family RNA methyltransferase | SUB1273 | 2002, 2021, 2053, 2055, 2062 | VMHJH_07695, VMHJH11_07695, VMHJH2_03260, BAUJH3_07800, BAUJH4_07825 |
| *lbP* | LPXTG cell wall anchor domain containing protein/ lactoferrin binding protein | SUB0145 | 2002, 2021, 2053, 2055, 2062 | VMHJH_06815, VMHJH1_07125, VMHJH2_0665, BAUJH3_05800, BAUJH4_06245 |
| *leuS* | Leucine tRNA-ligase | SUB1729 | 2002, 2021, 2053, 2055, 2062 | VMHJH_02825, VMHJH1_02825, VMHJH2_04945, BAUJH3_03415, BAUJH4_04720 |
| *Lmb* | Zinc ABC transporter substrate-binding protein | SUB0884 | 2002, 2021, 2053, 2055, 2062 | VMHJH_00790, VMHJH1_00790, VMHJH2_01315, BAUJH3_00790, BAUJH4_00790 |
| *mga/ vru* | Helix-turn-helix domain-containing protein | SUB0144 | 2002, 2021, 2053, 2055, 2062 | VMHJH_06810, VMHJH1_07130, VMHJH2_06655, BAUJH3_05795, BAUJH4_06250 |
| *mtsB* | Metal ABC transporter ATP-binding protein | SUB0474 | 2002, 2021, 2053, 2055, 2062 | VMHJH_04785, 04850, 07950, BAUJH3_04845, BAUJH4_02755 |
| *mtuA* | Metal ABC transporter substrate-binding protein | SUB0473 | 2002, 2021, 2053, 2055, 2062 | VMHJH_04780, VMHJH1_04855, VMHJH2_07955, BAUJH3_04850, BAUJH4_02760 |
| *oppF* | Putative oligopeptide permease/ ATP-binding cassette domain-containing protein | SUB1621 | 2002, 2021, 2053, 2055, 2062 | VMHJH_03370, VMHJH1_03370, VMHJH2_04425, BAUJH3_02870, BAUJH4_04175 |
| *pauA*/ *skc* | Streptokinase precursor | SUB1785 | 2002, 2021, 2053, 2055, 2062 | VMHJH_2530, VMHJH1_2 530, VMHJH2_05210, BAUJH3_03710, BAUJH4_05015 |
| *perR* | Peroxide responsive transcriptional repressor PerR | SUB1714 | 2002, 2021, 2053, 2055, 2062 | VMHJH_02900, VMHJH1_02900, VMHJH2_04870, BAUJH3_03340, BAUJH4_04645 |
| *purH* | Purine biosynthesis (IMP cyclohydrolase (EC 3.5.4.10) / Phosphoribosylaminoimidazolecarboxamide  formyltransferase (EC 2.1.2.3) | SUB0030 | 2002, 2021, 2053, 2055, 2062 | VMHJH_09285,VMHJH1_ 09315, VMHJH2_10120, BAUJH3_09220, BAUJH4_09305 |
| *purN* | Purine biosynthesis (Phosphoribosylglycinamide formyltransferase (EC 2.1.2.2) | SUB0029 | 2002, 2021, 2053, 2055, 2062 | VMHJH_09280, VMHJH1_09320, VMHJH2_10115, BAUJH3_09225, BAUJH4_09300 |
| Putative fructan beta-fructosidase precursor |  | SUB0135 | 2002, 2021, 2053, 2055, 2062 | VMHJH_06755, VMHJH1_07185,  VMHJH2_06700, BAUJH3_05740, BAUJH4_06305 |
| Putative surface-anchored 2′,3′-cyclic-nucleotide 2′-Phosphodiesterase |  | SUB0241 | 2002, 2021, 2053, 2055, 2062 | VMHJH_7245, VMHJH1_6695, VMHJH2_06145, BAUJH3_06235, BAUJH4_05810 |
| Putative surface-anchored protein |  | SUB0207 | 2002, 2021, 2053, 2055, 2062 | VMHJH_07090, VMHJH1_06850, VMHJH2_6285, BAUJH3_06080, BAUJH4_05965 |
| Putative surface-anchored protein |  | SUB0888 | 2002, 2021, 2053, 2055, 2062 | VMHJH_00770, VMHJH1_00770, VMHJH2_01355, BAUJH3-00770, BAUJH4_00770 |
| putative surface-anchored protein |  | SUB1730 | 2002, 2021, 2053, 2055, 2062 | VMHJH_02820, VMHJH1_02820, VMHJH2_04950, BAUJH3_03420, BAUJH4_04725 |
| Putative surface-anchored subtilase family protein |  | SUB0826 | 2002, 2021, 2053, 2055, 2062 | VMHJH_01055, VMHJH1_01055, VMHJH2_01050, BAUJH3_01055, BAUJH4_01055 |
| Putative zinc carboxypeptidase |  | SUB1370 | 2002, 2021, 2053, 2055, 2062 | VMHJH_08180, VMHJH1_08180, VMHJH2_09255, BAUJH3_08285, BAUJH4_08310 |
| *scpA* | S8 family Serine peptidase | SUB1154 | 2002, 2021, 2053, 2055, 2062 | VMHJH_05790, VMHJH1_06155, VMHJH2_02730 , BAUJH3_07245, BAUJH4_07270 |
| *scaR* | Metal-dependent transcriptional regulator | SUB0472 | 2002, 2021, 2053, 2055, 2062 | VMHJH_04775, VMHJH1_04860, VMHJH2_07960, BAUJH3_04855, BAUJH4_02765 |
| *srtA* | Sortase A | SUB0881 | 2002, 2021, 2053, 2055, 2062 | VMHJH_00805, VMHJH1_00805, VMHJH1_01300, BAUJH3_00805, BAUJH4_00805 |
| *sua*/ SUAM protein | Streptococcus uberis adhesion molecules | SUB1635 | 2002, 2021, 2053, 2055, 2062 | VMHJH_03305, VMHJH1_03305, VMHJH2_04490, BAUJH3_02935, BAUJH4_04240 |
| Uberolysin | Carnocyclin family circular bacteriocin | SUB0032 | 2002, 2021, 2055, 2062 | VMHJH_09295, VMHJH1_09305, BAUJH3_09210, BAUJH4_09315 |

*Putative virulence genes/ factors absent in the genomes studied are - *neuD*/ *epsM*, cfu_CAMP factor, CAMP_factor_family_pore-forming_toxin, *cps4I*, *legI*/ *neuB*, *wbjC*/ *cps4K*, *cpsL*, *neuA_1*, *hylB*, *wbpI*/ *cps4L*, *capD*/ *cps4J*, *neuC*, *cps4H*, *epsF*/ *cps4H*, *cps4G*, *cps4G_2*, *srtC*, *tagU_3*/ *cps4A*, *cps4E*, *emm*
